# Supplementary material for: Hollow Bioelectrodes Based on Buckypaper Assembly. Application to the Electroenzymatic Reduction of O2
Source: Nanomaterials (Basel). 2022 Jul 14;12(14):2399. doi: 10.3390/nano12142399 (PMC9317853; doi:10.3390/nano12142399)
Supplement: Supplementary file 1 [file nanomaterials-12-02399-s001.zip › nanomaterials-1795881-supplementary.pdf]

Supplementary Material

Hollow Bioelectrodes based on Buckypaper Assembly. Application to the Electroenzymatic Reduction of O_2

Paulo Henrique M. Buzzetti, Anastasiia Berezovska, Yannig Nedellec, Serge Cosnier*

Univ. Grenoble Alpes, CNRS, DCM, 38000 Grenoble, France
*serge.cosnier@univ-grenoble-alpes.fr

Additional Results

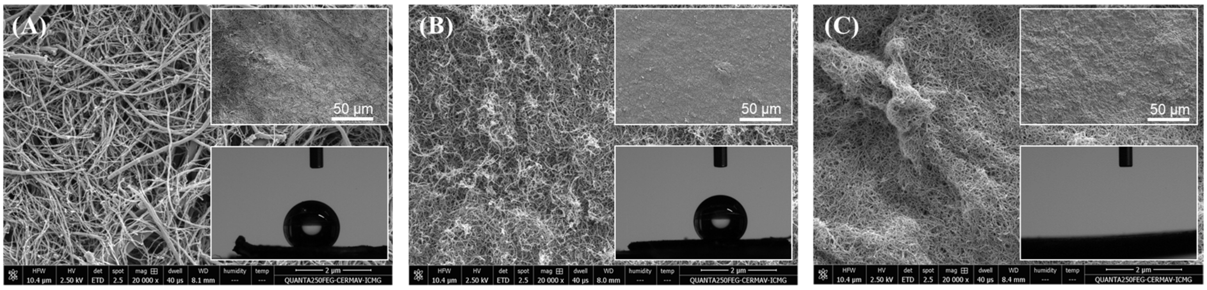

Figure S1. SEM images of A) C_{bp}, B) L_{bp} and C) hemin-L_{bp} and the respective water contact angles.

Figure S2 shows a single reversible system at $E_{1/2}$= -0.140 V vs RHE, corresponding to the electroactivity of hemin. A linear dependence of oxidative and reductive peak currents towards the scan rate is observed. This reflects an adsorption-controlled process for hemin corroborating its good adsorption on the MWCNT walls of L_{bp}.

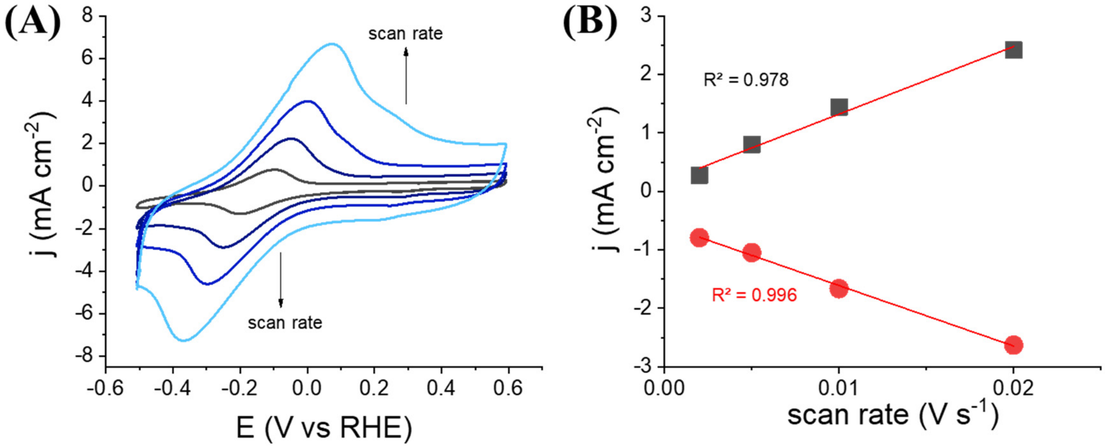

Figure S2. (**A**) Cyclic voltammograms recorded in 0.1 M phosphate buffer (pH 6.5) under argon for the hemin-L_{bp} at different scan rates from 2 to 20 mV s^{-1}. (**B**) Evolution of oxidation and reduction peak current towards scan rate with its respective coefficient of determination (R^2).
